# Intra-abdominal pressure, intra-abdominal hypertension, and pregnancy: a review

**DOI:** 10.1186/2110-5820-2-S1-S5

**Published:** 2012-07-05

**Authors:** Rosaleen Chun, Andrew W Kirkpatrick

**Affiliations:** 1 Department of Anesthesia, Foothills Medical Centre, University of Calgary, 1403-29th St. NW, Calgary, T2N 2T9, Canada; 2 Regional Trauma Services Program, Foothills Medical Centre, Alberta Health Services, 1403-29th St. NW, Calgary, T2N 2T9, Canada; 3 Department of Surgery, Foothills Medical Centre, Alberta Health Services, 1403-29th St. NW, Calgary, T2N 2T9, Canada; 4 Department of Critical Care Medicine, Foothills Medical Centre, Alberta Health Services, 1403-29th St. NW, Calgary, T2N 2T9, Canada

**Keywords:** intra-abdominal pressure, intra-abdominal hypertension, pregnancy, preeclampsia-eclampsia.

## Abstract

The last several decades have seen many advances in the recognition and prevention of the abdominal compartment syndrome (ACS) and its precursor, intra-abdominal hypertension (IAH). There has also been a relative explosion of knowledge in the critical care, trauma, and surgical populations, and the inception of a society dedicated to its understanding, the World Society of the Abdominal Compartment Syndrome (WSACS). However, there has been almost no recognition or appreciation of the potential presence, influence, and management of intra-abdominal pressure (IAP), IAH, and ACS in pregnancy. This review highlights the importance and relevance of IAP in the critically ill parturient, the current lack of normative IAP values in pregnancy today, along with a review of the potential relationship between IAH and maternal diseases such as preeclampsia-eclampsia and its potential impact on fetal development. Finally, current IAP measurement guidelines are questioned, as they do not take into account the gravid uterus and its mechanical impact on intra-vesicular pressure.

## Review

### Introduction

Despite the nearing deadline for attaining the World Health Organization's Millennium Development Goals that include improving maternal health worldwide by 2015, the reality remains that over one-half million expectant or new mothers die suddenly and unpredictably [1,2]. Although 90% of these deaths occur in developing countries, intensivists in developed nations are also confronted by unexpected critical illnesses in pregnancy, often in otherwise previously healthy women. These illnesses, most commonly preeclampsia and obstetric hemorrhage, can result in significant morbidity and mortality in both mother and newborn [3,4]. Further complicating the situation, intensivists are often unfamiliar with maternal-fetal physiology both in health and in critical illness [3], and perhaps do not consider the possible impact of intra-abdominal pressure (IAP) and intra-abdominal hypertension (IAH) on such conditions.

Since the inception of the World Society of the Abdominal Compartment Syndrome (WSACS) in 2004, many advances in the recognition, the treatment, and especially the prevention of the abdominal compartment syndrome (ACS) have occurred [5,6]. When clinicians are vigilant and make efforts to detect and treat raised IAP, it appears that deadly ACS may often be avoided, especially with the adoption of newer resuscitation strategies [7,8]. Such advances have led some to propose that the future efforts of the WSACS should be focused on the more prevalent but less understood precursor condition of IAH, rather than the overtly catastrophic ACS. This rapid evolution in practice in the fields of critical care and trauma has been associated with a relative explosion in the published world literature, focusing on critical care, trauma, medical, and surgical populations [9].

However, despite seminal work early in the twentieth century by an obstetrician, Paramore [10], there has been almost no recognition or appreciation of the potential presence, influence, and management of IAH in pregnancy and the peripartum state, other than dramatic case reports in which ACS was only recognized in a parturient *in extremis *[11-13]. As pregnancy is a natural but dramatic physiologic state that can occur at some point in the lifetime of approximately half of the world's population, further understanding and research is urgently needed. Critical illness in pregnancy, unfortunately, is not uncommon, given that the population-based incidence of severe obstetric morbidity has been reported to be as high as 1.2% in the UK [14]. In the USA, the American College of Obstetricians and Gynecologists published an overall estimate that critical care services were required in 1% to 3% of pregnant women [4], in whom diagnoses of maternal hypertension and hemorrhage were the most cited causes of critical care admission [4]. The relevance of greater understanding and education in both the behavior and measurement of the IAP in pregnancy is illustrated by the following case, aspects of which, primarily relating to the infectious etiology of the case, have previously been described [15].

### Illustrative case

A 16-year-old female, estimated to be at 32 weeks gestation, was transferred to a tertiary intensive care unit (ICU) from a peripheral hospital. She required intubation and ventilation following a 2-week prodrome of progressive cough. Her ICU admission revealed severe hypoxemia with a PaO_2_/FiO_2 _ratio of 53, despite receiving 100% fractional inspired oxygen (FiO_2_). Her initial lactate was 2.1 mmol/l, hemoglobin was 10.5 g/dl, and creatinine was 58 μmol/l with a urine output of greater than 30 ml/h. Her physiologic data can be found in Table 1. A fetal ultrasound revealed a poor biophysical profile score of 2/8; however, under conditions of maternal sedation and pharmacologic paralysis, this was non-diagnostic in determining fetal well-being. There was a normal amniotic fluid volume and a fetal heart rate of 156 beats/min. As a result, the obstetrical consult regarding the issue of fetal delivery was noncommittal, and the patient was inadvertently lost to follow up by obstetrics during her subsequent ICU course.

**Table 1:** Physiologic variables of reported case

Admission	Prior to laparotomy
MAWP = 44.5 mmHg	MAWP = 50 mmHg
SpO_2 _= 83%	SpO_2 _= 80%
FiO_2 _= 100%	FiO_2 _= 100%
7.32/52/53/26 BE = 0 mmol/l	7.23/83/55/33 BE = 4 mmol/l
UO = 27 to 185 ml/h	UO = 0 ml/h
CVP = 14 mmHg	CVP = 24 mmHg
No inotrope support	Norepinephrine = 0.06 μg/kg/min

MAWP, mean airway pressure; SpO_2_, pulse oximetry; FiO_2_, fractional inspired oxygen; BE, base excess; UO, urine output; CVP, central venous pressure.

The patient was ventilated using high-frequency oscillatory ventilation in combination with nitric oxide (NO), but the oxygenation did not improve significantly. Within 15 h of admission, bilateral chest tubes were required for presumed barotrauma-related pneumothoraces. She became anuric in association with a rising creatinine of 122 μmol/l. Nine hours later, she further decompensated, requiring an increase of the mean airway pressure (MAWP) to 50 cmH_2_O. The central venous pressure (CVP) increased to 27 mmHg, and severe subcutaneous emphysema developed 'from head to toe'. Transthoracic echocardiography revealed a hyperdynamic, underfilled heart. She continued to deteriorate, prompting a consult to cardiac surgery for extracorporeal life support (ECLS) as a last resort to her spiraling physiologic decline.

Obstetrics was re-consulted and noted that sonographic windows during a repeat fetal ultrasound were completely absent. After much debate, it was decided that cannulation for ECLS would precede a caesarean section (CS). In the operating room, the ECLS cannulation procedure proved difficult and prolonged, resulting in iatrogenic injuries to both the groin and subclavian vessels, and transfusion of six units of red blood cells. Finally, after cannulation, the ECLS circulatory flows were inadequate at 1 to 1.5 l/min.

Thereafter, the CS was performed by a midline laparotomy. Upon incising the peritoneum, an unexpected gush of air was released. There was an immediate improvement in mechanical ventilation; pulse oximetry (SpO_2_) increased above 90% for the first time since her admission. The ECLS flows only improved partially. The fetus was successfully delivered within 20 min. Upon evacuation of the uterus, there was a distinct improvement in the patient's ventilation and oxygenation. The ECLS flows immediately improved to normal levels (4 to 4.5 l/min); SpO_2 _was 100%; and the NO and jet ventilator were quickly weaned. There was a spontaneous diuresis of clear urine intraoperatively. The neonate was intubated after delivery, immediately taken to the neonatal intensive care unit, and eventually discharged on day 27 but had multiple hospital admissions thereafter.

Postoperatively, the inciting etiology of fulminant adult respiratory distress syndrome (ARDS) was determined to be caused by a severe influenza A infection, prompting publication of a plea for routine vaccination of all eligible patients with the influenza vaccine, although no mention of the potential role of ACS was made [15]. The mother herself underwent 2 weeks of ECLS support, repeated upper gastrointestinal bleeding due to systemic heparinization, the administration of 98 units of blood products, and several ECLS circuit thromboses. Although no preoperative IAP measurements had been obtained, postoperative IAP was 14 mmHg. She was discharged home 6 weeks later.

The evidence to support the conclusion that ACS significantly contributed to her spiraling decline included the high MAWP of 50 mmHg, a well-described inciting factor for tension pneumoperitoneum [16-22]. The progressive worsening of her ventilatory parameters, anuria, and elevated CVP in the face of a normal cardiac function, almost completely relieved by the release of a large 'gush' of air during laparotomy, strongly supported the contention that her ARDS was complicated by an unrecognized but classic presentation of fulminant ACS [23,24].

### The physiology of normal pregnancy

As IAH/ACS impacts almost all of the body's organ systems, it is important for intensivists to understand the normal alterations in these systems that occur with pregnancy. The pregnant state involves a complex and remarkable interplay of both adaptive and supportive physiology, allowing a fetus to grow and thrive for a finite duration *in utero *until such time it is ready for a physically independent life. The maternal physiologic changes that occur in pregnancy are multisystemic and far-reaching, not the least of which is the adaptation to accommodate the gravid uterus. On average, the uterus contributes 1 kg to the overall weight gain in pregnancy, while the amniotic fluid, fetus, and placenta comprise approximately 5 kg in additional weight [25].

To accommodate this growth, the thoracic cage increases in both anteroposterior and transverse diameters [25]. The hormone relaxin, released by the corpus luteum and placenta, results in targeted softening of the ligamentous structures to also compensate for uterine growth [26]. The diaphragm becomes elevated as a result of being pushed cephalad by the uterus, impeding the functional residual capacity by at least 20% [25]. Tidal volume increases and is associated with a 45% increase in minute and alveolar ventilation [25]. Overall maternal metabolic rate, oxygen consumption (VO_2_), gas exchange, and acid/base balance are all affected by several factors including the growth of the feto-placental unit, progesterone levels, and carbon dioxide production. On average, maternal VO_2 _increases by 15% to 20% [27], although in one study, VO_2 _increased with advancing gestation by 28% [28]. With increased ventilation, the resulting respiratory alkalosis is renally compensated through a reduction of serum bicarbonate to 20 mEq/L and total buffer base capacity to 5 mEq/L [25]. Thus, when critically ill, the parturient is more vulnerable to hypoxemia and acidemia, with overall less physiologic reserve, than when nonpregnant [27].

In addition, there is a 50% increase in plasma volume resulting in dilutional anemia and overall increase in circulating blood volume of 40% [25,27]. Cardiac output increases by 30% to 50%; blood flow to the gravid uterus increases tenfold [27]. After 20 weeks of gestation, the uterus size can cause a mechanical aorto-caval obstruction while fully supine and can result in the 'supine-hypotensive syndrome': significant loss of venous return for which the cardiovascular system cannot compensate [25]. However, the majority of women develop collateral circulation through interosseus vertebral, paravertebral, epidural, and ovarian venous systems [25]. It has been suggested that those who suffer from supine-hypotensive syndrome likely do not develop adequate collateral circulation [29]. While only approximately 8% of women at term experience this life-threatening situation, significant compression of the inferior vena cava (IVC) while supine does occur in the majority of women [25,29]. Whether elevated IAP can exacerbate aorto-caval compression and has a relationship with this syndrome is unknown. Thus, due to the myriad of hormonal, mechano-physiologic changes, the majority of parturients are well compensated for the exponential growth of their fetus in a relatively short duration of time.

### IAP and pregnancy

Knowledge of normal versus pathologic IAPs for any population in the ICU would seem intuitive for patient care [30]. To date, however, there is very little actual data regarding physiologic and pathophysiologic IAP in pregnancy. Current consensus guidelines group pregnancy and morbid obesity together as chronically compensated states of IAH [31]. However, the growth of each gestational age during pregnancy as well as the unique anatomic impact as the uterus grows from the pelvis into the abdominal cavity has never been taken into account. Unlike pregnancy, chronic obesity is the deposition of fat diffusely throughout the abdominal cavity. The implication of this anatomical difference could be considered semantic by some, but could be considered significant, given that the standard of measurement of IAP uses the intra-vesicular pressure as a surrogate; the location of which rests in the pelvis.

The state of the science in this regard is well symbolized by the fact that until very recently, the best evidence concerning IAP in pregnancy was obtained through rectal manometry on primarily primigravid inmates of an institution for 'fallen women' and published in 1913 [10]. More recently, however, Al-Khan and colleagues [32] published more contemporary IAP intravesical measurements in 100 healthy term parturients obtained under spinal anesthesia just prior to commencement of elective CS. These and all IAP data to date can be found in Table 2. They found the median IAP in a leftward tilted position to be 22 ± 2.9 mmHg (range 15 to 29 mmHg), pressures actually in the threshold range for ACS if organ failure were also present [31]. Postoperatively, after neonatal delivery, the IAP dropped significantly to a median IAP of 16 mmHg (range 11 to 24 mmHg) [32]. Besides questions regarding the fluid volume of bladder priming for IAP measurement and unknown spinal anesthesia dermatome distribution, the greatest question from this study is the unspecified degree of left lateral tilt during the IAP measurements, making it difficult to reconcile if these measurements reflected the actual abdominal IAP or the weight of the gravid uterus on the bladder itself.

**Table 2:** Physiologic IAP in pregnancy

Author	Year	*n*	Gestation	Positions during IAP measurement	IAP_mean_ (mmHg)	Comments
Paramore [10]	1913	24	6 months to term	Supine; left side; knee chest; standing	Range 15 to 44	Rectal manometer; ambulatory subjects
Cuppett et al. [62]	2008	40	Term	Supine; Left Lateral	Not reported	Elective CS under spinal anesthesia
Sugerman [49]	2011	5	39 weeks	Supine; Left Lateral decubitus	25 ± 3; 23 ± 3	Unclear methods; likely ambulatory patients
Al-Khan et al. [32]	2011	100	36 to 41 weeks	Leftward tilt	22 ± 2.9	Elective CS; Unspecified leftward tilt; 50 ml saline instilled in bladder; unclear reference point
Chun et al. [33]	2012	20	38 to 40 weeks	Supine; Leftward tilt	10 ± 4.7 8.9 ± 4.9	Elective CS under spinal anesthesia; leftward tilt 10^0^

IAP_mean_, mean intra-abdominal pressure; *n*, number; CS, caesarean section.

We similarly measured the IAP in 20 term parturients under spinal anesthesia [33]. The IAP measurement was significantly higher in the fully supine position (0°) compared to when the operating table was leftward tilted to 10° with the reference point held constant by placing the bladder pressure transducer in a line adjacent to the patient on an intravenous pole. We thus hypothesized that the weight of the gravid uterus might have directly impacted on the bladder, thereby falsely elevating the IAP measurement when fully supine.

Thus, the above studies highlight currently unresolved issues regarding the necessary trade-offs between the use of a standardized and reproducible reference position to obtain meaningful IAP data, and the reality of patient safety. This is akin to the concern regarding the positioning of ventilated patients fully supine to measure IAP, while increasing aspiration risks [30,34,35]. Left lateral tilt has become the standard of care in CS, particularly after spinal anesthesia, as a means to both facilitate CS and alleviate potential aorto-caval compression while supine [36]. There is debate as to the degree of tilt required to minimize compression of the IVC by the uterus [36,37]; a tilt of 15° is generally recommended [36]. What remains unknown is the effect of the gravid uterus on measured bladder pressure in varying relative positions to each other. Questions arise as to the validity of the IAP measurement as recommended by the WSACS guidelines in a pregnant patient from early second trimester onwards. Current recommended guidelines describe IAP measurement in the fully supine position at end-expiration [38,39]. Such a maneuver in pregnancy, however, could be detrimental. Clearly, more studies are needed to validate IAP measurement in this unique population.

### The potential role of IAH in preeclampsia-eclampsia

Preeclampsia, part of a spectrum of hypertensive disorders of pregnancy, is defined as the development of arterial hypertension and proteinuria after 20 weeks gestation [40] and is associated with significant maternal morbidity and death [4,40]. Preeclampsia-eclampsia, clinically, can present with one or more manifestations of either renal compromise, neurological sequelae including visual disturbances, headache, stroke, and eventually convulsions (eclampsia), to thrombocytopenia, fetal growth restriction, and liver and other hematologic abnormalities [41]. HELLP is considered a severe variant of preeclampsia and manifests as a syndrome of hemolysis, elevated liver enzymes, and low platelet count [41]. While some of these clinical manifestations, particularly eclampsia, have been well described as early as the mid-1600's [42], modern understanding of the etiology remains incomplete. The most commonly held hypothesis is that abnormal placentation occurs during the myometrial trophoblastic invasion in the second trimester [40], leading to placental ischemia and the release of angiogenic toxins, causing widespread endothelial dysfunction [41] and generalized inflammation. However, this immune maladaptation hypothesis has been recently questioned [43] as a result of recent epidemiologic studies. While its incidence worldwide is significant (3% to 5% of all pregnancies) [40], preeclampsia is a heterogeneous condition for which its commoner presentation in younger women in developing countries may be etiologically distinct than that of the somewhat older preeclamptic presentation in developed nations, with clinically [30] milder disease occurring later in gestation [43].

Two dramatic case reports described overt ACS as a complication of preeclampsia-eclampsia/HELLP syndromes requiring urgent life-saving interventions [12,13]. The diagnosis of peripartum ACS in these cases was challenging not only due to the lack of well-established normative pregnant values of IAP, but also because of the overlap of signs and symptoms between ACS and severe preeclampsia such as oliguria and nonspecific abdominal pain [12]. Furthermore, we contend that ACS was unrecognized in these cases because the routine measurement of IAP generally has not been accepted in many ICUs. Akin to many other conditions in critical illness, clinicians too frequently do not consider the possible impact of IAH in the clinical picture, especially when the patient has not been injured or subjected to surgery [44,45].

Even as early as the 1900s, investigators had suggested uncompensated elevated IAP as a possible etiologic factor in the development of preeclampsia [10,46]. While elevated IAP may not be the only, sole, or critical inciting factor, its potential role in the development or progression of such syndromes certainly is plausible. Mauriceau noted the preponderance of 'toxemia' of pregnancy in primiparas in 1694 [42]. Paramore, also noting this prevalence, hypothesized that nulliparous and muscular women were prone to spastic abdominal wall tone resulting in elevated IAPs, compromising perfusion pressure to the abdominopelvic viscera [10,46]. Mulier was able to indeed confirm a linear relationship between abdominal pressure and volume to calculate the abdominal wall elastance (*E*) [47] and even found that *E *decreased significantly with increased age and gravidity [48]. Sugerman recently hypothesized that IAH played a central role in initiating the multi-system cascade of diminished perfusion and inflammation associated with the various clinical manifestations of preeclampsia [49]. He speculated obstructed venous return from IAH, essentially limiting abdominal perfusion pressure due to increased back pressure, resulted in decreased end-organ perfusion including both kidneys and the placenta. Thus, the activation of the renin-angiotensin system, with elevation of aldosterone levels, systemic hypertension, and placental ischemia/necrosis with an impact on fetal growth, was triggered [49].

That ACS occurs in this patient population is not really the question. Given the evidence in the literature to date (Table 1), it is likely that term pregnancy is associated with elevated IAP to which the patient has adapted. It is also likely that the IAP is elevated in the immediate postpartum phase as well [32,50], similar to the postoperative surgical populations [51]. What remains to be seen is whether preeclamptic patients truly have IAH, at what pressure does this occur, and whether IAH has a significant role the in the progression of the development of severe preeclampsia or HELLP.

### IAH and the fetus

Despite the limited understanding of IAH in maternal care, even less is known regarding its effects on the fetus. Whether there are subclinical effects of even modest elevations of maternal IAP on the fetus is completely unknown. Several animal studies have confirmed that the mammalian fetus *in utero *is subject to transmitted IAP [52,53]. IAH was found to decrease uterine blood flow and induce a resultant compensatory fetal hypertension [54] such as during laparoscopy even with inert gasses rather than CO_2_. In a gravid rabbit model, Karnak et al. examined the relationship between maternal IAP and intra-amniotic pressure (IAMNP) through catheters inserted into both the intraperitoneal and intra-amniotic cavities at 20 days of gestation. Intraperitoneal air was insufflated to an IAP of 20 cmH_2_O. They found that IAMNP was linearly related to IAP as defined by IAMNP = IAP × 0.8 + 2.0. Further, they found that the elevation of IAMNP to 15.6 cmH_2_O via the elevation of the IAP (to 17 cmH_2_O) altered the contractile properties of the fetal bladder [53].

While we are aware of no modern human data correlating maternal IAH with any known effects on the fetus, concerns regarding the fetal-placental unit are neither entirely novel nor implausible. Tanyel [55] hypothesized that elevated IAMNP is translated to elevated fetal IAP, both of which were vulnerable to elevations in maternal IAP [55]. Through this mechanism, elevated fetal IAP could result in increased urethral resistance, the chronicity of which could lead to abnormal development of the bladder detrusor muscles, resultant dysfunctional voiding in children, and possible urinary tract anomalies [55]. Although the etiology of such syndromes is likely multifactorial, exploration of the impact of pathological maternal IAH on the feto-placental unit could be another area of fruitful potential investigation.

### Ovarian hyperstimulation syndrome

Ovarian hyperstimulation syndrome (OHSS) is a not an uncommon complication of ovulation induction for assisted reproduction [56,57]. The mechanism is not entirely understood but is thought to be mediated by vasoactive cytokines in response to exogenous administration of human chorionic gonadotropin [58]. Significant third spacing as a result of capillary vascularity due to ovarian neoangiogenesis can occur [56], and in its most severe form, massive and rapid accumulation of abdominal ascites results in an overt ACS [58]. Management for this condition ranges from conservative observation to intensive care admission with IAP monitoring and paracentesis to relieve ACS [57,58]. As assisted reproduction increases in prevalence, it becomes imperative to recognize this relatively common complication and to consider the potential role of IAH in its pathophysiology.

Like OHSS, rapid growth in abdominal girth, dyspnea, abdominal pain, and other overt symptoms of ACS in other gynecological conditions must also be considered in the differential. Patients undergoing ovulation induction are also at increased risk of ovarian torsion and ectopic pregnancy [57]. Meigs' syndrome, solid ovarian tumors associated with hydrothorax and ascites, has been described similarly to OHSS in presenting with symptoms of ACS [59]. However, while OHSS is often self-limited with conservative management as a viable option, definitive therapy for Meigs' syndrome would be surgical removal of the tumor itself [59].

## Conclusions

It is currently a recommended standard for any newly admitted critically ill patient with any two IAH risk factors to have baseline IAP measured [38]. The critically ill pregnant patient typically has positive generic risk factors for IAH such as 'acute respiratory failure with elevated intrathoracic pressures' and 'increased abdominal contents' in later pregnancy, in addition to those specific to their inciting illness. If IAP is not measured, IAH will often be missed. Further, the lack of knowledge of the behavior of IAH in pregnancy risks the potential disaster of missed or delayed diagnosis of ACS resulting in morbidity and mortality in a relatively young and otherwise healthy cohort. Despite these high stakes, there is almost no data to guide evidence-based decisions. At the bedside, measuring the IAP and considering IAH in all critical maternal conditions is essential, especially in preeclampsia-eclampsia where some have hypothesized that IAH may have an additional role. Research is urgently needed to define the normal range of IAP in all phases of pregnancy. This may be better facilitated with the validation of less invasive IAP measurement alternatives such as the measurement of wall tension [60] or via gastric tonometry [61]. The IAP in pregnancy must take into account the precautions for aorto-caval compression. The potential impact of maternal IAH on fetal development is essentially unknown. Finally, IAH leading to ACS is a real and potential complication in early induced pregnancy. Measurement of the IAP should be performed in the management of OHSS.

## Abbreviations

ACS: abdominal compartment syndrome; CS: caesarean section; CVP: central venous pressure; ECLS: extracorporeal life support; FiO_2_: fractional inspired oxygen; IAH: intra-abdominal hypertension; IAMP: intra-amniotic pressure; IAP: intra-abdominal pressure; ICU: intensive care unit; MAWP: mean airway pressure; OHSS: ovarian hyperstimulation syndrome; SpO_2_: pulse oximetry; VO_2_: oxygen consumption; WSACS: World Society of the Abdominal Compartment Syndrome.

## Competing interests

Dr. Andrew W Kirkpatrick is the principal investigator of an ongoing randomized trial of vacuum therapy in open-abdomen management in critical illness/injury that is funded by the KCI Corporation, although there is no personal benefit to him. He is the Chairman of the Guidelines Committee of the World Society of the Abdominal Compartment Syndrome. Dr. Rosaleen Chun declares that she has no competing interests.

## Authors' contributions

RC drafted the original manuscript; was responsible for the conception, design, the data acquisition, analysis and interpretation of this original work; as well as revision for important intellectual content. AWK made substantial contributions to the final manuscript, the data acquisition, analysis, and interpretation of this work; as well as critically revised this original work for important intellectual content. Both authors read and approved the final manuscript.

## References

[B1] World Health Organization The World Health Report 2005-Make Every Mother and Child Count. Geneva 2005

[B2] United Nations The Millenium Development Goals Report 2010. New York 2010

[B3] Zeeman GG Obstetric critical care: a blueprint for improved outcomes Crit Care Med 2006 34 S208 214 10.1097/01.CCM.0000231884.99763.69 16917425

[B4] American College of Obstetricians and Gynecologists ACOG Practice Bulletin No. 100: critical care in pregnancy Obstet Gynecol 2009 113 443 450 19155919 10.1097/AOG.0b013e3181993087

[B5] Cheatham ML Safcsak K Is the evolving management of intra-abdominal hypertension and abdominal compartment syndrome improving survival? Crit Care Med 2010 38 402 407 10.1097/CCM.0b013e3181b9e9b1 20095067

[B6] Cheatham ML Safcsak K Intra-abdominal hypertension and abdominal compartment syndrome: the journey forward Am Surg 2011 77 1 5 21944444

[B7] Balogh ZJ Martin A van Wessem KP King KL Mackay P Havill K Mission to eliminate postinjury abdominal compartment syndrome Arch Surg 2011 146 938 943 10.1001/archsurg.2011.73 21502442

[B8] Cotton BA Au BK Nunez TC Gunter OL Robertson AM Young PP Predefined massive transfusion protocols are associated with a reduction in organ failure and postinjury complications J Trauma 2009 66 41 48 10.1097/TA.0b013e31819313bb 19131804

[B9] Balogh ZJ van Wessem K Yoshino O Moore FA Postinjury abdominal compartment syndrome: are we winning the battle? World J Surg 2009 33 1134 1141 10.1007/s00268-009-0002-x 19343417

[B10] Paramore RH The intra-abdominal pressure in pregnancy Proc R Soc Med 1913 6 291 334 19977092 10.1177/003591571300600964 PMC2006436

[B11] Soltsman S Russo P Greenshpun A Ben-Ami M Abdominal compartment syndrome after laparoscopic salpingectomy for ectopic pregnancy J Minim Invasive Gynecol 2008 15 508 510 10.1016/j.jmig.2008.04.010 18602054

[B12] Richter CE Saber S Thung SF Eclampsia complicated by abdominal compartment syndrome Am J Perinatol 2009 26 751 753 10.1055/s-0029-1223289 19444766

[B13] Dart BW IV Cockerham WT Torres C Kipikasa JH Maxwell RA A novel use of recombinant factor VIIa in HELLP syndrome associated with spontaneous hepatic rupture and abdominal compartment syndrome J Trauma 2004 57 171 174 10.1097/01.TA.0000135142.80368.65 15284569

[B14] Waterstone M Bewley S Wolfe C Incidence and predictors of severe obstetric morbidity: case-control study BMJ 2001 322 1089 1093 10.1136/bmj.322.7294.1089 11337436 PMC31259

[B15] Parkins MD Fonseca K Peets AD Laupland KB Shamseddin K Gill MJ A potentially preventable case of serious influenza infection in a pregnant patient CMAJ 2007 177 851 853 10.1503/cmaj.070622 17923651 PMC1995144

[B16] Ali SZ Freeman BD Coopersmith CM Abdominal compartment syndrome in a patient resulting from pneumothorax Intensive Care Med 2003 29 1614 10.1007/s00134-003-1903-z 12879231

[B17] Andrew TA Milne DD Pneumoperitoenum associated with pneumothorax or pneumopericardium: a surgical dilemma in the injured patient Injury 1979 11 65 70 10.1016/S0020-1383(79)80131-X 521144

[B18] Glauser FL Bartlett RH Pneumoperitoneum in association with pneumothorax Chest 1974 66 536 540 10.1378/chest.66.5.536 4279163

[B19] Quek R Lim ST Tan EH Pneumoperitoneum following percutaneous lung biopsy Lancet 2006 368 1794 10.1016/S0140-6736(06)69739-0 17113429

[B20] Ralston C Clutton-Brock TH Hutton P Tension pneumoperitoneum Intensive Care Med 1989 15 532 533 10.1007/BF00273567 2607041

[B21] Winer-Muram H Rumbak MJ Bain RSJ Tension pneumoperitoneum as a complication of barotrauma Crit Care Med 1993 21 941 943 10.1097/00003246-199306000-00026 8504664

[B22] Larsson A Clinical significance of elevated intraabdominal pressure during common conditions and procedures Acta Clin Belg Suppl 2007 74 77 17469704

[B23] Schein M Wittmann DH Aprahamian CC Condon RE The abdominal compartment syndrome: the physiological and clinical consequences of elevated intra-abdominal pressure J Am Coll Surg 1995 180 745 753 7773495

[B24] Pelosi P Quintel M Malbrain ML Effect of intra-abdominal pressure on respiratory mechanics Acta Clin Belg Suppl 2007 Suppl 1 78 88 24881704 10.1179/acb.2007.62.s1.011

[B25] Gaiser R Chestnut D, Polley L, Tsen L, Wong C Physiologic changes of pregnancy Chestnut's Obstetric Anesthesia: Principles and Practice 2009 4 Philadelphia: Mosby Elsevier 15 26

[B26] Bani D Relaxin: a pleiotropic hormone Gen Pharmacol 1997 28 13 22 10.1016/S0306-3623(96)00171-1 9112071

[B27] Suresh MS LaToya Mason C Munnur U Cardiopulmonary resuscitation and the parturient Best Pract Res Clin Obstet Gynaecol 2010 24 383 400 10.1016/j.bpobgyn.2010.01.002 20418169

[B28] Highman TJ Friedman JE Huston LP Wong WW Catalano PM Longitudinal changes in maternal serum leptin concentrations, body composition, and resting metabolic rate in pregnancy Am J Obstet Gynecol 1998 178 1010 1015 10.1016/S0002-9378(98)70540-X 9609576

[B29] Scott DB Kerr MG Inferior vena caval pressure in late pregnancy J Obstet Gynaecol Br Commonw 1963 70 1044 1049 10.1111/j.1471-0528.1963.tb15051.x 14100067

[B30] De Keulenaer BL De Waele JJ Powell B Malbrain ML What is normal intra-abdominal pressure and how is it affected by positioning, body mass and positive end-expiratory pressure? Intensive Care Med 2009 35 969 976 10.1007/s00134-009-1445-0 19242675

[B31] Malbrain ML Cheatham ML Kirkpatrick A Sugrue M Parr M De Waele J Balogh Z Leppaniemi A Olvera C Ivatury R D'Amours S Wendon J Hillman K Johansson K Kolkman K Wilmer A Results from the International Conference of Experts on Intra-abdominal Hypertension and Abdominal Compartment Syndrome. I. Definitions Intensive Care Med 2006 32 1722 1732 10.1007/s00134-006-0349-5 16967294

[B32] Al-Khan A Shah M Altabban M Kaul S Dyer KY Alvarez M Saber S Measurement of intraabdominal pressure in pregnant women at term J Reprod Med 2011 56 53 57 21366128

[B33] Chun R Baghirzada L Kirkpatrick A Measurement of intra-abdominal pressure in term pregnancy: a pilot study Int J Obstet Anesth 2012 21 135 139 10.1016/j.ijoa.2011.10.010 22326198

[B34] McBeth PB Zygun DA Widder S Cheatham M Zengerink I Glowa J Kirkpatrick AW Effect of patient positioning on intra-abdominal pressure monitoring Am J Surg 2007 193 644 647 10.1016/j.amjsurg.2007.01.013 17434374

[B35] Cheatham ML De Waele JJ De Laet I De Keulenaer B Widder S Kirkpatrick AW Cresswell AB Malbrain M Bodnar Z Mejia-Mantilla JH Reis R Parr M Schulze R Puig S World Society of the Abdominal Compartment Syndrome (WSACS) Clinical Trials Working Group The impact of body position on intra-abdominal pressure measurement: a multicenter analysis Crit Care Med 2009 37 2187 2190 10.1097/CCM.0b013e3181a021fa 19487946

[B36] Kinsella SM Lateral tilt for pregnant women: why 15 degrees? Anaesthesia 2003 58 835 836 10.1046/j.1365-2044.2003.03397.x 12911353

[B37] Bamber JH Dresner M Aortocaval compression in pregnancy: the effect of changing the degree and direction of lateral tilt on maternal cardiac output Anesth Analg 2003 97 256 258 12818977 10.1213/01.ane.0000067400.79654.30

[B38] Cheatham ML Malbrain ML Kirkpatrick A Sugrue M Parr M De Waele J Balogh Z Leppaniemi A Olvera C Ivatury R D'Amours S Wendon J Hillman K Wilmer A Results from the International Conference of Experts on Intra-abdominal Hypertension and Abdominal Compartment Syndrome. II. Recommendations Intensive Care Med 2007 33 951 962 10.1007/s00134-007-0592-4 17377769

[B39] Malbrain ML Cheatham ML Definitions and pathophysiological implications of intra-abdominal hypertension and abdominal compartment syndrome Am Surg 2011 77 6 11 21944445

[B40] Contreras F Fouillioux C Bolivar A Betancourt MC Colmenares Y Rivero M Israili ZH Velasco M Endothelium and hypertensive disorders in pregnancy Am J Ther 2003 10 415 422 10.1097/00045391-200311000-00007 14624279

[B41] Silasi M Cohen B Karumanchi SA Rana S Abnormal placentation, angiogenic factors, and the pathogenesis of preeclampsia Obstet Gynecol Clin North Am 2010 37 239 253 10.1016/j.ogc.2010.02.013 20685551

[B42] Chesley LC History and epidemiology of preeclampsia-eclampsia Clin Obstet Gynecol 1984 27 801 820 10.1097/00003081-198412000-00004 6396011

[B43] Dekker G Robillard PY Pre-eclampsia: is the immune maladaptation hypothesis still standing? An epidemiological update J Reprod Immunol 2007 76 8 16 10.1016/j.jri.2007.03.015 17493684

[B44] Ball CG Kirkpatrick AW McBeth P The secondary abdominal compartment syndrome: not just another post-traumatic complication Can J Surg 2008 51 399 405 18841232 PMC2556543

[B45] Biffl WL Moore EE Burch JM Offner PJ Franciose RJ Johnson JL Secondary abdominal compartment syndrome is a highly lethal event Am J Surg 2001 182 645 648 10.1016/S0002-9610(01)00814-5 11839331

[B46] Paramore RH Eclampsia and its incidence [abstract] Proc R Soc Med 1922 15 14 16 19982406 10.1177/003591572201500912 PMC2102267

[B47] Mulier JP Dillemans M Crombach C Missant C Sels A On the abdominal pressure volume relationship The Internet Journal of Anesthesiology 2009 21 1

[B48] Mulier JP Dillemans B Heremans L Determinants of the abdominal pressure volume relation in non ACS patients Acta Clin Belg Suppl 2007 62 289

[B49] Sugerman HJ Hypothesis: preeclampsia is a venous disease secondary to an increased intra-abdominal pressure Med Hypotheses 2011 77 841 849 10.1016/j.mehy.2011.07.051 21862236

[B50] Abdel-Razeq SS Campbell K Funai EF Kaplan LJ Bahtiyar MO Normative postpartum intraabdominal pressure: potential implications in the diagnosis of abdominal compartment syndrome Am J Obstet Gynecol 2010 203 149 e1-4 20417482 10.1016/j.ajog.2010.02.055

[B51] Sanchez NC Tenofsky PL Dort JM Shen LY Helmer SD Smith RS What is normal intra-abdominal pressure? Am Surg 2001 67 243 248 11270882

[B52] Attah AA Hutson JM The role of intra-abdominal pressure in cryptorchidism J Urol 1993 150 994 996 8102185 10.1016/s0022-5347(17)35672-0

[B53] Karnak I Aksoz E Ekinci S Onur R Tanyel FC Increased maternal intraabdominal pressure alters the contractile properties of fetal rabbit bladder J Pediatr Surg 2008 43 1711 1717 10.1016/j.jpedsurg.2008.01.025 18779012

[B54] Curet MJ Weber DM Sae A Lopez J Effects of helium pneumoperitoneum in pregnant ewes Surg Endosc 2001 15 710 714 10.1007/s004640000390 11591973

[B55] Tanyel FC Urinary tract anomalies and dysfunctional voiding: a spectrum dictated by the influence of amniotic pressure upon fetal urodynamics Med Hypotheses 2000 54 140 145 10.1054/mehy.1998.0819 10790740

[B56] Kumar P Sait SF Sharma A Kumar M Ovarian hyperstimulation syndrome J Hum Reprod Sci 2011 4 70 75 10.4103/0974-1208.86080 22065820 PMC3205536

[B57] Madill JJ Mullen NB Harrison BP Ovarian hyperstimulation syndrome: a potentially fatal complication of early pregnancy J Emerg Med 2008 35 283 286 10.1016/j.jemermed.2007.11.074 18403169

[B58] Chen CD Wu MY Chao KH Lien YR Chen SU Yang YS Update on management of ovarian hyperstimulation syndrome Taiwan J Obstet Gynecol 2011 50 2 10 10.1016/j.tjog.2011.01.014 21482366

[B59] Peparini N Di Matteo FM Silvestri A Caronna R Chirletti P Abdominal hypertension in Meigs' syndrome Eur J Surg Oncol 2008 34 938 942 10.1016/j.ejso.2007.08.006 17905563

[B60] van Ramshorst GH Salih M Hop WC van Waes OJ Kleinrensink GJ Goossens RH Lange JF Noninvasive assessment of intra-abdominal pressure by measurement of abdominal wall tension J Surg Res 2011 171 240 244 10.1016/j.jss.2010.02.007 20462598

[B61] Malbrain ML Different techniques to measure intra-abdominal pressure (IAP): time for a critical re-appraisal Intensive Care Med 2004 30 357 371 10.1007/s00134-003-2107-2 14730376

[B62] Cuppett C Wilson A Janoo J Bringman J Toffle R Effect of BMI on intraabdominal pressure measurements in the third trimester of pregnancy Am J Obstet Gynecol 2008 199 S151

